# A Systematic Review of Glucose Transport Alterations in Alzheimer's Disease

**DOI:** 10.3389/fnins.2021.626636

**Published:** 2021-05-20

**Authors:** Natalia Kyrtata, Hedley C. A. Emsley, Oli Sparasci, Laura M. Parkes, Ben R. Dickie

**Affiliations:** ^1^Division of Neuroscience and Experimental Psychology, Faculty of Biology, Medicine and Health, Manchester Academic Health Science Centre, The University of Manchester, Manchester, United Kingdom; ^2^University Hospitals of Morecambe Bay NHS Foundation Trust, Lancaster, United Kingdom; ^3^Lancaster Medical School, Lancaster University, Lancaster, United Kingdom; ^4^Department of Neurology, Lancashire Teaching Hospitals NHS Foundation Trust, Preston, United Kingdom; ^5^Greater Manchester Mental Health NHS Foundation Trust, Manchester, United Kingdom; ^6^Geoffrey Jefferson Brain Research Centre, Manchester Academic Health Science Centre, Manchester, United Kingdom

**Keywords:** GLUT 3, GLUT 1, blood-brain barrier, glucose transporters, Alzheimer's disease

## Abstract

**Introduction:** Alzheimer's disease (AD) is characterized by cerebral glucose hypometabolism. Hypometabolism may be partly due to reduced glucose transport at the blood-brain barrier (BBB) and across astrocytic and neuronal cell membranes. Glucose transporters (GLUTs) are integral membrane proteins responsible for moving glucose from the bloodstream to parenchymal cells where it is metabolized, and evidence indicates vascular and non-vascular GLUTs are altered in AD brains, a process which could starve the brain of glucose and accelerate cognitive decline. Here we review the literature on glucose transport alterations in AD from human and rodent studies.

**Methods:** Literature published between 1st January 1946 and 1st November 2020 within EMBASE and MEDLINE databases was searched for the terms “glucose transporters” AND “Alzheimer's disease”. Human and rodent studies were included while reviews, letters, and *in-vitro* studies were excluded.

**Results:** Forty-three studies fitting the inclusion criteria were identified, covering human (23 studies) and rodent (20 studies). Post-mortem studies showed consistent reductions in GLUT1 and GLUT3 in the hippocampus and cortex of AD brains, areas of the brain closely associated with AD pathology. Tracer studies in rodent models of AD and human AD also exhibit reduced uptake of glucose and glucose-analogs into the brain, supporting these findings. Longitudinal rodent studies clearly indicate that changes in GLUT1 and GLUT3 only occur after amyloid-β pathology is present, and several studies indicate amyloid-β itself may be responsible for GLUT changes. Furthermore, evidence from human and rodent studies suggest GLUT depletion has severe effects on brain function. A small number of studies show GLUT2 and GLUT12 are increased in AD. Anti-diabetic medications improved glucose transport capacity in AD subjects.

**Conclusions:** GLUT1 and GLUT3 are reduced in hippocampal and cortical regions in patients and rodent models of AD, and may be caused by high levels of amyloid-β in these regions. GLUT3 reductions appear to precede the onset of clinical symptoms. GLUT2 and GLUT12 appear to increase and may have a compensatory role. Repurposing anti-diabetic drugs to modify glucose transport shows promising results in human studies of AD.

## Introduction

### Background

Alzheimer's disease (AD) is a chronic neurodegenerative disorder characterized by the presence of β-amyloid (Aβ) plaques and neurofibrillary tangles (NFTs) (Grundke-Iqbal et al., 1986; Scheltens et al., 2016). The majority of AD is sporadic, with <5% being classified as genetic (Reitz et al., 2011). Several mechanisms have been proposed for its pathophysiology. According to the amyloid cascade hypothesis, the Aβ precursor protein (APP) is abnormally cleaved, leading to an imbalance between Aβ production and clearance, favoring the accumulation of Aβ. This, in turn, forms clusters in the brain which induce oxidative stress, leading to synaptic dysfunction, neuronal death, and subsequent cerebral atrophy (Chételat et al., 2010). These clusters, called oligomers, form fibrils, then beta-sheets and eventually develop into plaques which are considered a hallmark of AD (Grundke-Iqbal et al., 1986). While Aβ accumulation has a critical role in AD, it is becoming increasingly recognized that brain Aβ burden does not correlate with the severity of cognitive impairment (Games et al., 1995; Price et al., 2009). Aβ accumulation also occurs in aging individuals without cognitive impairment (Castello and Soriano, 2014; Morris et al., 2014; Herrup, 2015), indicating the limitations of the amyloid hypothesis (Kametani and Hasegawa, 2018). The tau hypothesis conjectures that tau is the main causative protein for AD (Kosik et al., 1986). Tau is a protein normally associated with microtubules which serve to stabilize tubulin assemblies. In AD, tau is abnormally hyperphosphorylated and forms pathological inclusions known as NFTs, which are widely identified in AD brains. Tau is more strongly associated with cognitive impairment than Aβ (Hanseeuw et al., 2019). However, attempts to stabilize cognitive function through modification of Aβ and tau in the clinical setting have been unsuccessful to date (Congdon and Sigurdsson, 2018; Yiannopoulou and Papageorgiou, 2020).

### Hypometabolism in AD

In addition to Aβ and tau, AD is considered a metabolic disorder, which relates to reduced cerebral glucose metabolism, brain insulin resistance, and age-induced mitochondrial dysfunction (Van Der Velpen et al., 2019). The conventional view is that reduced brain metabolism is secondary to brain atrophy and neuronal loss (Bokde et al., 2001). However, there is accumulating evidence that hypometabolism occurs before the onset of brain atrophy and clinical symptoms, indicating that changes in metabolism may occur prior to reduced glucose demand by tissues (De Leon et al., 2001; Jagust et al., 2006; Mosconi et al., 2006, 2008, 2009; Masdeu, 2008).

Abnormal cerebral glucose metabolism was observed using FDG-PET in 1983 by de Leon et al. (1983), who observed a 17–24% reduction in the regional cerebral metabolic rate of glucose (*CMR*_glu_) in a cohort of 24 AD patients. This correlated with a reduction in cognitive performance compared to age matched controls. Later studies confirmed these results, building support for regional declines in *CMR*_glu_ as a hallmark of AD, including in frontal white matter, caudate, thalamus, temporal, and parietal regions (Small et al., 2000; Mosconi et al., 2004, 2007a). Masdeu (2008) examined seven pre-symptomatic, at-risk subjects with familial AD using MRI and FDG-PET imaging. Compared to seven matched healthy controls, the familial AD subjects showed a reduction in glucose metabolism in most brain regions examined, including the whole brain, right, and left inferior parietal lobules, superior temporal gyrus, left entorhinal cortex, posterior cingulate cortex, and hippocampus. De Leon et al. (2001), Mosconi et al. (2008), and Jagust et al. (2006) examined *CMR*_glu_ in multiple brain regions of healthy elderly individuals using FDG-PET and MRI scans to differentially predict cognitive decline from normal aging. In all three studies, reductions in glucose metabolism predicted cognitive decline in those participants who went on to develop cognitive impairment or a clinical diagnosis of AD.

In a longitudinal study, Mosconi et al. (2009) examined four cognitively normal elderly subjects and three patients with mild AD. Their pathological diagnosis was verified through post-mortem studies 6 ± 3 years after the subjects' last FDG-PET scan. All participants who were cognitively intact at baseline developed mild cognitive impairment (MCI) 2–7 years after their baseline assessment. On post-mortem studies, two of these subjects had definite AD, one had probable AD and the last had pathological findings consistent with Parkinson's disease with mild AD-related pathology. In all four patients, *CMR*_glu_ was reduced in areas involving the hippocampus, up to 7 years before their diagnosis of MCI. Follow up FDG-PET scans showed progressive reductions in *CMR*_glu_ with wider brain involvement including the temporal and parietal lobes, and to varying degrees the anterior and posterior cingulate regions. A more recent study by Ou et al. (2019) examining 551 participants with AD observed reduced metabolism as measured using FDG-PET as an independent biomarker for AD. Their findings demonstrate faster cognitive decline and brain atrophy in participants with reduced metabolism.

### Glucose Transport as the Rate-Limiting Step in Glucose Metabolism

While these studies included a small number of participants, their results suggest that hypometabolism precedes a clinical diagnosis of AD. Glucose metabolism requires both delivery of glucose to cells from the bloodstream, and phosphorylation by hexokinase at the site of mitochondria. One possible explanation for early changes to glucose metabolism observed via FDG-PET may be due to abnormal delivery of glucose to the brain. Glucose is a hydrophilic molecule and requires transporters to cross cell membranes. Glucose uptake into the brain occurs predominantly via the sodium-independent facilitative transporters GLUT1 and GLUT3, encoded by the SLC2A1 and SLC2A3 genes, respectively. GLUT1 is responsible for glucose uptake across the BBB endothelial cells, where the higher density isoforms are located (55 kDa), and into astrocytes, where the lower density isoforms are located (45 kDa). Glucose uptake into the brain appears to correlate with the number of GLUT1 transporters at the BBB (Zeller et al., 1997). Neurons do not express GLUT1 (Zlokovic, 2011). The main glucose transporter that facilitates uptake of glucose into neurons is GLUT3, which is encoded by the SLC2A3 gene. GLUT3 is also detected at lower levels on astroglial and endothelial cells (Kumari and Heese, 2010; Patching, 2017). Low levels of GLUT2, encoded by the SLC2A2 gene, are present on astrocytes (Magistretti and Pellerin, 1999; Larrabee, 2002). Reduced supply of glucose to the brain via loss of these major glucose transporters may lead to a brain glucose deficit, halting metabolism and other processes dependent on ATP production (Iadecola, 2015).

**Figure 1:**
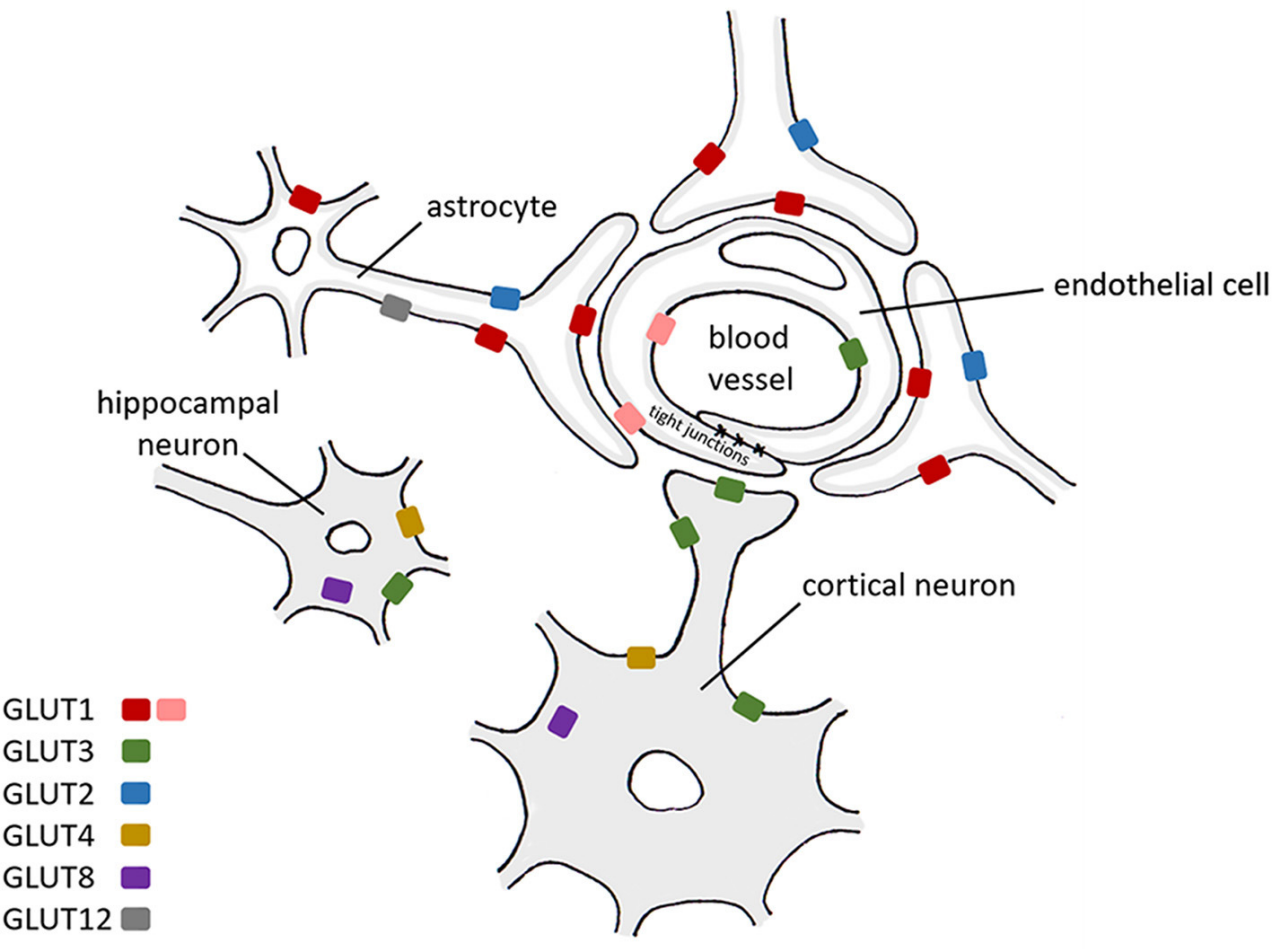
A schematic diagram representing the expression of glucose transporters within CNS cells. Red: GLUT1 55 kDa isoform, pink: GLUT1 45 kDa isoform. GLUT1 (Kumari and Heese, 2010), GLUT2 (Kumari and Heese, 2010), GLUT3 (Kumari and Heese, 2010), GLUT4 (Leloup et al., 1996; Vannucci et al., 1998; Reagan, 2002; Kumari and Heese, 2010), GLUT8 (Kumari and Heese, 2010), GLUT12 (Zhang et al., 2014) (details extracted from https://www.brainrnaseq.org on 19/04/2021).

Several insulin-sensitive transporters are present in the brain at low levels, including GLUT4, a transporter mainly present in non-cerebral fat and muscle tissue, GLUT8, which has been suggested to contribute toward glucose homeostasis in hippocampal neurons (Piroli et al., 2002), and GLUT12, a newly discovered glucose transporter found primarily in insulin-sensitive tissues (Szablewski, 2017). The location of these transporters within the central nervous system (CNS) and their presence on different cell types are shown in Table 1, Figure 1.

**Table 1:** GLUTs expressed in the central nervous system.

**Protein (*gene*)**	**References**	**Insulin sensitive (Yes/No)**	**Site expressed in the CNS**
GLUT1 (*SLC2A1*)	Kumari and Heese, 2010	No	Endothelial cells (55 kDa isoforms), astrocytes (45 kDa isoforms)
GLUT2 (*SLC2A2*)	Kumari and Heese, 2010	No	Astrocytes
GLUT3 (*SLC2A3*)	Kumari and Heese, 2010	No	Endothelial cells, astrocytes, hippocampal neurons
GLUT4 (*SLC2A4*)	Leloup et al., 1996; Vannucci et al., 1998; Reagan, 2002; Kumari and Heese, 2010	Yes	Hypothalamic neurons, hippocampal neurons, cerebellar neurons, sensorimotor cortex, pituitary
GLUT8 (*SLC2A8*)	Kumari and Heese, 2010	Yes	Hypothalamic neurons, hippocampal neurons
GLUT12 (*SLC2A12*)	Zhang et al., 2014 (details extracted from https://www.brainrnaseq.org)	Yes	Cortical astrocytes

Detailed descriptions of glucose transporters and their respective functions are not described here—these aspects have been reviewed extensively elsewhere by Szablewski (2017) and Koepsell (2020). Szablewski (2017) also reviewed glucose transporter alterations in AD with particular focus on links between AD and insulin resistance, but a systematic review of human and animal data was not performed. Furthermore, Szablewski did not cover in detail results from tracer studies of glucose uptake into the brain, the effects of AD pathologies such as amyloid-β and tau on GLUTs, the timing of effects, or how GLUT alterations affect the brain. Here we perform a systematic review to capture all human and rodent studies of glucose transport alterations in AD to date and aim to evaluate the evidence to support (i) which transporters are affected, if any, (ii) how glucose uptake into the brain is altered, (iii) which brain regions are most affected, (iv) when changes occur relative to other AD pathologies, and (v) how GLUT changes affect brain function.

## Methods

**Figure 2:**
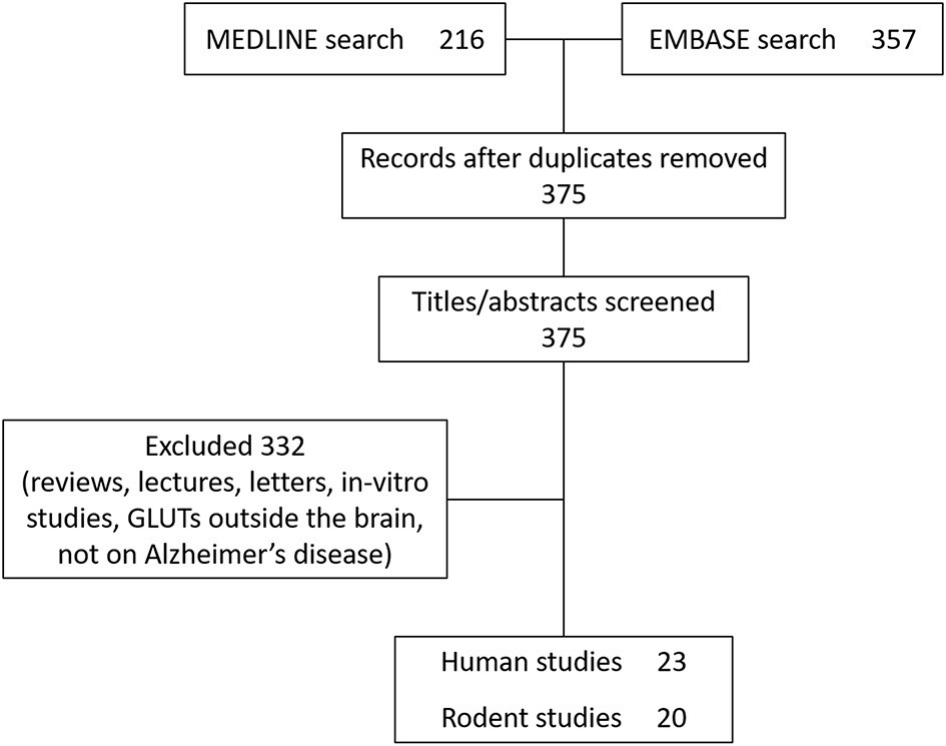
PRISMA diagram.

Literature published between 1st January 1946 and 1st November 2020 was searched using the PubMed search engine. Exploded headings were used for “glucose transporters” and “Alzheimer's disease” with the Boolean operator “AND.” Searches were performed in EMBASE and MEDLINE databases and duplicates were removed. Only studies investigating cerebral glucose transporters were included. Titles and abstracts were scanned to identify relevant papers and articles, and those which did not clearly examine glucose transport specific to Alzheimer's disease were discussed with the research team and excluded if not considered relevant. Human and rodent studies (post-mortem and *in-vivo*) were included and results are summarized in Tables 2, 3. Reviews, letters and *in-vitro* studies were excluded. Figure 2 shows the PRISMA flow chart detailing the search results.

**Table 2:** A summary of results from human post-mortem and tracer studies.

**References**	**Methods**	**Findings**
Kalaria and Harik, 1989	Immunohistochemistry study of brain tissue obtained from the frontal and temporal neocortex, hippocampus, putamen, cerebellum, and cerebral microvessels in AD subjects and controls to determine levels of hexose transporter (likely GLUT1, although not specified).	Significant reduction in hexose expression (likely GLUT1) transporter in brain microvessels, cerebral neocortex and hippocampus of AD brain.
Kawai et al., 1990	Immunohistochemistry study of the relationship between Aβ plaques, capillary density (collagen-4), and glucose transporters (GLUT1).	Collagen-4 and GLUT1 expression was reduced within Aβ plaques and increased in the immediate surroundings of Aβ plaques relative to gray matter.
Harik and Kalaria, 1991	Immunohistochemistry study of GLUT1 in cerebral microvessels of subjects with AD and age matched controls using irreversible binding to the ligand [3H] cytochalasin B.	GLUT1 expression was decreased by ~50% in cerebral microvessels of patients with AD compared to age-matched controls.
Harik, 1992	Immunohistochemistry study of GLUT1 in AD brain tissue and controls.	Reduction in the expression of GLUT1 in the cerebral microvessels in AD brain compared to age-matched controls, with no change in the density of GLUT1 in the erythrocyte membranes.
Horwood and Davies, 1994	Immunohistochemistry study of AD brain tissue obtained from the central part of the hippocampal formations (dentate gyrus, cornu ammonis, and subicular complex) to determine levels of microvascular GLUT1.	GLUT1 expression was significantly reduced in the microvessel endothelium in hippocampi of AD subjects compared to controls.
Simpson et al., 1994	Immunoblotting study of brain tissue obtained from AD subjects and controls.	Reduced glucose metabolism in the temporal and parietal regions of AD subjects. Reduced expression of GLUT1 and GLUT3 in the cerebral cortex of AD brains compared to controls, with greater and more significant reductions in GLUT3.
Harr et al., 1995	Immunohistochemistry study of brain tissue obtained from the dentate gyrus of AD subjects' to determine levels of GLUT3.	Significant reduction (49.5%) in GLUT3 expression in the outer portion of the molecular layer of the dentate gyrus in AD brains.
Mooradian et al., 1997	Western-blot study of GLUT1 in brain tissue from AD subjects and controls.	GLUT1 expression was reduced in AD but GLUT1 mRNA concentrations were not significantly different.
Liu et al., 2008	Western-blots and immune-dot-blot study of GLUT1-4 levels in the frontal cortex of frozen human brain tissue in subjects with AD and controls.	Decreased expression of GLUT1 and GLUT3 in AD brain tissue which correlated to hyperphosphorylation of tau and neurofibrillary tangle density. Downregulation of hypoxia-inducible factor 1 (HIF-1) in AD brain.
Liu et al., 2009	Western-blots and immune-dot-blot study of brain tissue obtained by autopsy from AD patients, subjects who had T2DM and subjects who had both AD and T2DM.	GLUT1 expression was significantly lower in AD brains. GLUT2 expression was significantly higher in AD brain and brain of subjects with both AD and T2DM. GLUT3 expression was significantly lower in all three groups with the lowest levels in T2DM brain. O-GlcNAcylation of global proteins and tau was downregulated in T2DM brain and AD brain. Tau phosphorylation is higher in T2DM brain and AD brain.
Wang et al., 2012	A case-control study was performed in a Chinese population of 597 patients with AD and 605 healthy controls examining the role of *SLC2A14*, the gene encoding GLUT14, in developing late-onset AD. Results were stratified by ApoEε4-carrying status.	The rs10845990 polymorphism within the gene coding for GLUT14 was significantly associated with late onset AD in non-ApoEε4 allele carriers (*p* < 0.001).
Pujol-Gimenez et al., 2014	Western blot study of brain tissue obtained from the frontal cortex of AD subjects and age-matched controls measuring the expression of GLUT12.	GLUT12 expression was significantly increased in AD compared to age-matched controls.
Burke et al., 2014	Immunohistochemistry study of GLUT1 in patients with AD, vascular dementia and patients who had suffered from stroke. The cumulative vessel length and diameter of hippocampal microvessels was measured using stereological spherical probe software.	Increases in percentage per area were found in GLUT1 density in AD cases in the: • CA1 of the hippocampus compared with post-stroke non-demented subjects (*p* = 0.011). • CA1 of the hippocampus compared with post-stroke demented subjects (*p* = 0.037). • CA2 of the hippocampus compared to vascular dementia subjects (*p* = 0.04). • Entorhinal cortex compared with post-stroke non-demented subjects (*p* = 0.004).
		Post-stroke demented cases had significantly lower vascular length than AD (*p* = 0.016). Post-stroke non-demented cases had significantly lower vascular length compared with controls (*p* = 0.015).
Mullins et al., 2017	Immunohistochemistry study into the relationship between brain insulin resistance and glucose transporter expression and the propensity to develop plaques and NFT.	Regional expression of GLUT1 showed a negative correlation with NFT density. Regional expression of GLUT4 showed a positive correlation with NFT density. Areas with reduced insulin signaling proteins (including IRS-1) showed higher NFT load.
An et al., 2018	LC-MS/MS study to measure GLUT1 and GLUT3 levels in brain tissue obtained from the middle frontal gyrus of 14 participants with AD, 14 controls, and 15 with asymptomatic AD (ASYMAD).	GLUT3 levels were lower in both AD and ASYMAD groups compared to controls, before and after adjusting sex, age at death and neuronal nuclear protein levels. Lower levels of GLUT3 correlated with the severity of both Aβ and NFT pathology.
Hase et al., 2019	Immunohistochemistry study of endothelial GLUT1. Microvascular pathology, capillary width and densities were measured using histopathological methods in the frontal lobe white matter across several dementia types including 18 participants with AD.	Collapsed string microvessels along with loss of GLUT1 immunoreactivity was detected in AD frontal lobe white matter compared to overlying cortex.
Friedland et al., 1989	Dynamic FDG-PET study in patients with probable Alzheimer's disease (AD) and healthy age-matched controls.	There was no significant difference in rate constants for glucose transport (*k_1_* and *k_2_*) or phosphorylation (*k_3_*).
Jagust et al., 1991	Dynamic FDG-PET study in six subjects with clinical criteria for probable AD and normal controls.	Decrease in *K_1_* in frontal and temporal cortex in AD subjects compared to controls, minimal differences in occipital cortex and white matter and decreased rCMR_glc_ in all cortical regions. Non-significant decrease in *k_3_* in all brain regions of AD subjects.
Kimura and Naganawa, 2005	Dynamic FDG-PET study in three subjects; a 45-year-old normal subject, a 65-year-old subject with mild AD, and a 70-year-old subject with severe AD.	Glucose transport was globally reduced in both AD cases compared to the normal subject. Glucose phosphorylation was diminished in gray matter of the severe case of AD, excluding the sensory, motor, and visual cortices. In the mild case, phosphorylation was reduced in the right parieto-temporal area.
Piert et al., 1996	Dynamic FDG-PET study in AD subjects and normal controls.	Significant reductions in glucose transport (*K_1_*) and phosphorylation (*k_3_*) in patients with AD compared to healthy age-matched controls in multiple cortical and subcortical regions of the brain with the greatest significant difference in the parietal and temporal cortex.
Mosconi et al., 2007b	Dynamic FDG-PET study with arterial blood sampling in 7 AD patients and 6 age matched controls and CMR_glc_ was calculated.	AD patients showed significant CMR_glc_ reductions in the hippocampus and posterior cingulate cortex. *K_1_* was reduced in the hippocampus and *k_3_* was reduced in the hippocampus, PCC and amygdala.
Gejl et al., 2017	FDG-PET study as part of a randomized control trial using 6 month treatment of GLP-1 analog or placebo in AD subjects, measuring blood-brain glucose transfer capacity (*T*_max_) and cerebral metabolic rate of glucose (CMR_glc_) in the AD patients and controls.	CMR_glc_ estimates were positively correlated with cognition while *T*_max_ and CMR_glc_ estimates were inversely correlated with AD duration. GLP-1 analog treatment significantly raised *T*_max_ estimates of cerebral cortex from 0.72 to 1.1 umol/g/min, matching *T*_max_ estimates in healthy volunteers.

**Table 3:** A summary of results from rodent post-mortem and tracer studies.

**References**	**Methods**	**Findings**
Ding et al., 2013	Longitudinal immunohistochemical and imaging study investigating hippocampal GLUT1, GLUT3, and GLUT4, glucose transport into the brain in female 3xTgAD mice (aged between 3 and 15 months).	Both 3xTgAD and wild-types underwent significant age-related reductions in glucose transport as detected using FDG-microPET, beginning at 6-9 months of age, but mechanisms were different. Reductions in GLUT1 (55 kDa isoform), increases in GLUT1 (45 kDa) and reductions in GLUT3 were observed in 3xTgAD mice, and non-monotonic changes in GLUT1 (55 kDa), decreases in GLUT3, and increases in membrane GLUT4 were observed in wild-types.
Do et al., 2014	Immunohistochemistry study investigating hippocampal GLUT1 expression in 3xTgAD mice aged between 3 and 18 months and APP/PS1 mice aged 8 months. Vascular volume fraction and uptake of D-glucose were measured using radionuclide-based brain perfusion tracers.	GLUT1 expression and D-glucose uptake were reduced in 18 month old 3xTgAD mice, but no differences were found in glucose transport or GLUT1 in either strain at 8 months. Reduced vascular volume fractions were observed at 6 months in 3xTg mice and in 8 month old APP/PS1 mice.
Griffith et al., 2019	Longitudinal analysis of 3xTg mice studied at ages 1–3, 6–8, and 16–18 months. Glucose tolerance was assessed alongside Western blot analysis of hippocampal insulin pathways PI3K/AKT and MAPK/ERK, and glucose transporters GLUT3 and GLUT4. GLUT1 was not studied.	Glucose tolerance and plasma insulin levels were found to be reduced as early as 1 month, well before detection of plaques (14 months). GLUT3 reductions but not GLUT4 were observed later at 18–20 months.
Hooijmans et al., 2007	Immunohistochemistry study into the causal relationship between GLUT1 reductions in the hippocampus and cortex and Aβ. Computer-assisted analysis of capillary density, and Aβ in young (8 months) and old (18 months) APP/PS1 mice. GLUT1 expression was normalized to capillary density to correct for potential loss of vascular volume.	At 8 months, GLUT1, capillary density or GLUT1 amount per capillary density were not different between APP/PS1 and wild-types. At 18 months, GLUT1 was reduced in the hippocampus of 18 month APP/PS1 mice relative to wild-types, while capillary density was not significantly different. The ratio of GLUT1 amount per capillary density was decreased in the dendate gyrus only. Aging produced significant reductions in GLUT1 and capillary density, but the ratio of GLUT1 amount per density was unchanged with age. No cortical changes were observed.
Kouznetsova et al., 2006	Immunohistochemistry study of Tg2576 mice aged 4–18 months to stain cortical GLUT1 and Aβ plaques and to assess impact of size and load of Aβ plaques on GLUT1 expression.	GLUT1 was reduced in cortical regions with high plaque load was associated with greater reductions compared to areas with low plaque load. Around large plaques, the capillary density was lower than around diffuse smaller plaques.
Kuznetsova and Schliebs, 2013	Immunohistochemistry study investigating GLUT1 and Aβ in somatosensory cortex of Tg2576 mice aged between 4 and 18 months.	No changes in GLUT1 at 10 months, when plaque deposition is beginning, but reductions found in 18 month mice when plaque load is considerable indicating Aβ may contribute to reductions in GLUT1.
Gil-Iturbe et al., 2020	Western blot study investigating GLUT1, GLUT3, GLUT4, and GLUT12 in the frontal cortex of two amyloidogenic mouse models Tg2576 (16 months old) and APP/PS1 (16 months old). Age effects assessed in C57/6/SJL wild-type mice aged 2–3 and 18 months old. To assess effects of Aβ directly, Aβ1–42 was injected intra-cerebroventricularly in 3 month old C47BL/6J mice.	Tg2576 and APP/PS1 mice exhibited decreased GLUT1 and GLUT3 and increased GLUT12. No changes were found in GLUT4. No age-dependent effects were found in GLUT1 and GLUT3 in C57/6/SJL mice. In Aβ1–42 injected mice, the same patterns in up- and downregulation of GLUTs were observed indicating a direct link between GLUTs and amyloid toxicity.
Merlini et al., 2011	Immunohistochemistry study into the morphology, biochemistry and functionality of cortical and hippocampal blood vessels in arcAβ mice at 6, 9–12, and 16–22 months. GLUT1 and GLUT3 measured by immunoblot, corrected for vascular volume using CD31. Brain glucose levels dynamically measured following i.v. administration of glucose using microdialysis.	Reductions in BBB and astrocytic GLUT1, but not GLUT3, reduced from mid-stage pathology onwards. Glucose uptake as measured using microdialysis confirmed reduced glucose transport. IgG extravasation observed at late-stages.
Ahn et al., 2018	Immunohistochemistry study investigating GLUT1 and tight junction protein ZO-1 expression in 4.5 and 9 month old 5xFAD mice.	Reductions in GLUT1 and ZO-1 observed in the hippocampus and cortex at both ages, which got worse with age and correlated with worsening of astrocyte activation (GFAP) and amyloid deposition.
Shang et al., 2019	Immunohistochemistry study assessing the impact of chronic cerebral hypo-perfusion (CCH) and AD pathology on cortical GLUT1 expression in 12 month old APP23 mice.	GLUT1 was reduced in cortex of APP23 mice compared to wild-types, which was further reduced in APP23 mice subject to CCH, indicating that AD and cerebrovascular pathologies may interact to exacerbate GLUT1 changes.
Lee et al., 2013	Western blot study of whole-brain expression of GLUT1, GLUT3, and GLUT4 and insulin markers in 12 month old NSE/hPS2m mice.	Reductions in GLUT1 and GLUT3, but not GLUT4 observed. Blood glucose found to be higher in AD mice.
Chua et al., 2012	Longitudinal western blot study of GLUT3 and GLUT4 AβPPsw/PS1ΔE9 mice	Reduced brain glucose and insulin content in 12 and 15 month old brains accompanied by increased GLUT3 and GLUT4, which preceded significant upregulation of Aβ42 in brain. GLUT1 not measured.
Deng et al., 2009	Western blotting study of insulin signaling and glucose transporters in intracerebroventricular streptozotocin (STZ) rat model of AD (6 months old, 21 days after STZ injections).	Reduced GLUT1 and GLUT3 in the cerebrum, and reduced GLUT3 in the cerebellum. Reduced pERK1 and pPI3K, pGSK-3β(S9) markers, and increased phosphorylation of tau.
Salkovic-Petrisic et al., 2014	Study investigating effect of long-term galactose administration on brain metabolism and glucose transporters via Western blot in intra-cerebroventricular streptozotocin (STZ) rat model of AD. Adult rats were used, and galactose administered for 1 month.	STZ rat exhibited significantly reduced GLUT3 expression in the hippocampus, which was normalized with galactose administration.
Knezovic et al., 2017	Western blot study investigating expression of GLUT2, insulin receptor, and neuroinflammatory marker GFAP in the hippocampus and cortex in intra-cerebroventricular streptozotocin (STZ) rat model of AD (adult males). Measurements were taken 1 h after STZ administration.	GLUT2 increased in the hippocampus, but unchanged in cortical regions. Insulin receptor decreased in parietal and temporal cortex, but not the hippocampus.
Biswas et al., 2018	Western blot and immunohistochemistry study investigating the expression of cortical and hippocampal GLUT1 and GLUT3, and brain glucose levels in intra-cerebroventricular streptozotocin (STZ) rat model of AD (adult males), and correlation with markers of endoplasmic reticulum stress, and astrocyte/microglia activation (Cd11b).	Glucose transporters GLUT1 and GLUT3 and brain glucose concentration were reduced in STZ rats. These changes were accompanied by reduced mitochondrial activity, increased endoplasmic reticulum stress, and increased microglial activation.

## Results

Twenty-three human studies and twenty rodent studies met the inclusion criteria. The transporter GLUT1 was most commonly investigated (*n* = 23), followed by GLUT3 (*n* = 13). A total of 10 human studies and 12 rodent studies observed reductions in GLUT1 expression, primarily in cortical and hippocampal regions. One study found increased GLUT1 expression. A total of 5 human studies and 6 rodent studies found reductions to GLUT3 expression. One study found no change, and one study found increased GLUT3 expression. There were fewer studies investigating GLUT2 (*n* = 2, both showing increased expression), GLUT4 (*n* = 6; no changes observed in 4 studies, one study showing increased GLUT4), GLUT12 (*n* = 1 showing increased expression), and GLUT14 (*n* = 1). Tracer-based methods were used to measure glucose uptake into brain tissue in 9 studies. All tracer studies except one reported reduced glucose uptake into the brain with AD, although in rodent studies reduced uptake was not observed until later stages of disease. A detailed review of all studies is given below.

### Evidence of Glucose Transporter Alterations in AD From Post-Mortem Human and Rodent Studies

Early work by Kalaria and Harik (1989) showed a significant reduction in hexose transporters, primarily GLUTs, in the neocortex and hippocampus of post-mortem AD brain tissue. In a subsequent study, Harik (1992) showed a significant reduction in the density of GLUT1 in the cerebral microvessels in the AD brain compared to age-matched controls, with no change in the density of GLUT1 in erythrocyte membranes. Simpson et al. (1994) showed that AD patients display reduced density of vascular and non-vascular forms of glucose transporters, GLUT1 and neuronal GLUT3. After correcting for synaptic loss, which is a prominent feature of AD (Masliah et al., 1990, 1991; Terry et al., 1991; Honer et al., 1992), the authors confirmed that the reduction in GLUT density persisted. Similar results were shown by Horwood and Davies (1994) who observed GLUT1 reductions in hippocampal tissue of the AD brain. Harik and Kalaria (1991) observed a decrease of ~50% in the density of glucose transporters in cerebral microvessels of patients with AD. They identified glucose transporters by reversible and irreversible binding to the ligand [3H] cytochalasin B. The type of glucose transporter identified using this method was not stated, however, cytochalasin B is an inhibitor of glucose transport in erythrocytes (May, 1988; Carruthers and Helgerson, 1991) and is likely to reflect GLUT1 levels.

Mooradian et al. (1997) observed a reduction in GLUT1 in the frontal and parietal cortex of AD brain, albeit with unchanged GLUT1 mRNA concentrations. In a study of hippocampal microvasculature, Burke et al. (2014) examined GLUT1 as a marker of capillary density in post mortem brain tissue of patients with AD, stroke, vascular dementia, and mixed-type dementia. Contrary to previous studies, they observed increased GLUT1 density in AD brain tissue compared to controls. By contrast, a very recent study investigating white matter tissue from the AD brain demonstrated collapsed string microvessels along with loss of GLUT1 immunoreactivity in the white matter of the frontal lobe compared to overlying cortex (Hase et al., 2019).

Liu et al. (2009) studied AD patients with and without type-2 diabetes mellitus (T2DM), and patients with T2DM alone. They showed that GLUT1 was significantly reduced in AD but not significantly reduced in either T2DM or T2DM-AD groups. GLUT3 was significantly reduced in all three groups with the lowest levels in T2DM brain. Interestingly, GLUT2 was significantly increased in the AD brain and brains of subjects with both AD and T2DM, possibly due to astrocyte overactivation (Liu et al., 2009). Harr et al. (1995) observed reductions in GLUT3 levels in the outer portion of the molecular layer of the dentate gyrus in AD brains.

A small number of studies have investigated the link between glucose transporters and AD pathologies in human tissue. Kawai et al. (1990) investigated the relationship between Aβ plaques and capillary glucose transporter density. Capillary glucose transporter density was reduced within Aβ plaques but increased in the immediate surroundings of Aβ plaques. Liu et al. (2008) found decreased levels of GLUT1 and GLUT3 in hippocampus and entorhinal cortex of AD brain tissue correlated with hyperphosphorylation of tau and NFT density. A recent study using participants from the Baltimore Longitudinal Study of Aging cohort measured GLUT1 and GLUT3 levels in the middle frontal gyrus of 14 participants with AD, 14 controls, and 15 with asymptomatic AD pathology, i.e., participants who exhibited significant AD pathology at post-mortem (including Aβ plaques, NFTs, and neuropil threads) but without evidence of cognitive dysfunction as assessed shortly before death. GLUT3 levels were significantly lower in both AD and asymptomatic AD groups relative to controls, before and after adjusting for sex, age at death, and neuronal nuclear protein levels. Lower levels of GLUT3 correlated with the severity of both Aβ and NFT pathology (An et al., 2018). GLUT1 levels were not significantly different in any of the groups.

Pujol-Gimenez et al. (2014) and Wang et al. (2012) investigated glucose transporters other than GLUT1, GLUT2, and GLUT3. Pujol-Gimenez et al. (2014) identified that the expression of GLUT12, a newly discovered glucose transporter found primarily in insulin-sensitive tissues (Stuart et al., 2009), was significantly increased in the frontal cortices of AD subjects compared to age-matched controls. Wang et al. (2012) examined the role of SLC2A14, the gene encoding GLUT14, in blood samples of patients with developing late onset AD. They performed a case-control study in a Chinese population of 597 patients with AD and 605 healthy controls, showing that SLC2A14 polymorphisms appear to confer increased risk of developing AD.

Alterations in GLUTs have also been observed in rodent models of AD. In a longitudinal study of 3xTgAD mice between 3 and 15 months of age, glucose transporters GLUT1 (55 and 45 kDa), and GLUT3 were found to change in both AD and wild-type animals but with differing temporal trajectories (Ding et al., 2013). In AD mice, GLUT1 (55 kDa) and GLUT3 were found to decrease with age, whereas GLUT1 (45 kDa) was found to increase with age. In wild-types, non-monotonic changes with age were observed for GLUT1 (55 kDa), whereas GLUT1 (45 kDa) was unchanged. GLUT3 decreased, and GLUT4 increased with age. Unfortunately, a formal comparison between glucose transporter expression between 3xTgAD mice and wild-types was not performed. In another longitudinal study, Do et al. (2014) studied vascular volume fraction and GLUT1 expression in the hippocampus of 3xTgAD and APP/PS1 mice and found no change in GLUT1 compared to wild-types at 8 months, but significantly reduced GLUT1 expression in 3xTgAD mice at 18 months after substantial amyloid-pathology had developed. APP/PS1 mice were not studied at this later timepoint. Griffith et al. (2019) investigated GLUT3 and GLUT4, but not GLUT1, longitudinally in 3xTg mice. They found similar timing of effects on GLUT3 as observed for GLUT1 by Do et al. (2014). Young rats exhibited reduced glucose tolerance (as early as 1 month), but GLUT3 did not change relative to wild-types until at least 18–20 months. GLUT4 was unaltered at all ages. Similar results were found in other AD models. Hooijmans et al. (2007) did not find any changes in GLUT1 expression between APP/PS1 mice and wild-types at 8 months, but found significantly reduced total GLUT1, reduced capillary density, and reduced GLUT1 per vascular volume fraction in 18 month old mice. Changes were found in the hippocampus, but not the cortex. In the Tg2576 model, Kuznetsova and Schliebs (2013) showed that cortical GLUT1 was unaltered at 10 months compared to wild-types, but at 18 months after development of amyloid pathology, AD mice had significantly lower cortical GLUT1.

A study by Kouznetsova et al. (2006) aimed to investigate if the degree of GLUT1 changes were related to amyloid load. In cortical regions with high amyloid load, GLUT1 staining was reduced compared to regions with low amyloid load. The authors also showed that GLUT1 staining was reduced nearer large senile plaques, relative to changes observed near smaller diffuse plaques. Gil-Iturbe et al. (2020) performed a more thorough investigation of GLUTs, measuring expression of GLUT1, GLUT3, GLUT4, and GLUT12 in two amyloidogenic models (Tg2576 and APP/PS1) aged 16 months old. They also investigated effects of aging in C57/6/SJL mice and the effects of amyloid on GLUTs via intracerebral injection of Aβ_1−42_. The authors found reduced GLUT1 and GLUT3, and increased GLUT12 in both strains. No age dependent effects on GLUTs were observed, conflicting with results from Ding et al. (2013). Injection of Aβ_1−42_ produced similar reductions in GLUT1 and GLUT3 as found in the Tg2576 and APP/PS1 mice, indicating a direct link between GLUT1 decreases and amyloid. Merlini et al. (2011) investigated GLUT changes in the arcAβ model. They observed reductions in BBB and astrocytic GLUT1 in the cortex and hippocampus from 9 to 12 months onward, which coincided with changes in glucose uptake as measured using microdialysis. However, GLUT3 was unaltered. By 16–22 months, IgG extravasation was observed, indicating loss of BBB integrity. In 5xFAD aged 4.5 and 9 months, reductions in GLUT1 and tight junction protein ZO-1 were found at both timepoints (Ahn et al., 2018). GLUT3 was not studied. Another study in 12 month old NSE/hPS2m mice also showed reductions in GLUT1 and GLUT3, but not GLUT4 (Lee et al., 2013). A study by Chua et al. (2012) in AβPPsw/PS1ΔE9 mice did not agree with changes observed in other models. Brain glucose levels were measured to be lower than wildtypes at 12 and 15 months of age, but GLUT3 and GLUT4 were found to be upregulated, not decreased (Chua et al., 2012). Unfortunately, changes to GLUT1 were not studied.

Shang et al. (2019) investigated the additional effects of chronic cerebral hypoperfusion on GLUT1 expression in APP23 mice. In normal APP23 mice, GLUT1 reductions were observed at 12 months, which were further reduced in APP23 mice with chronic cerebral hypoperfusion (Shang et al., 2019).

A number of studies have investigated GLUT expression in a rat model of AD produced by administering streptozotocin via intracerebroventricular injection. These studies found reduced expression of GLUT1 (Deng et al., 2009; Biswas et al., 2018), reduced expression of GLUT3 (Deng et al., 2009; Salkovic-Petrisic et al., 2014; Biswas et al., 2018), and increased expression of GLUT2 (Knezovic et al., 2017).

### Evidence of Altered Glucose Transport in AD From *in-vivo* Tracer Studies

Friedland et al. (1989) used FDG-PET to measure cerebral transport and phosphorylation rates of glucose in patients with probable AD and healthy age-matched controls. No difference in the transport rate constants, *K*_1_ and *k*_2_, or the utilization rate *k*_3_, were observed between groups. Two later FDG-PET studies in AD patients showed different results; Jagust et al. (1991) showed a reduction in the transport rate constant *K*_1_ in the frontal and temporal cortex with minimal differences in occipital cortex and total brain white matter, and a reduction in regional *CMR*_glu_ in all cortical regions compared to controls; Piert et al. (1996) found significant reductions in *K*_1_ and *k*_3_ in multiple cortical and subcortical regions of the brain in people with AD compared to controls with the most significant reductions being seen in the parietal and temporal cortices. Kimura and Naganawa (2005) performed dynamic PET studies in three subjects, a 45-year-old normal subject, a 65-year-old subject with mild AD, and a 70-year-old subject with severe AD. Glucose transport was globally reduced in both AD cases compared to the normal subject. Glucose phosphorylation was diminished in gray matter of the severe case of AD, excluding the sensory, motor, and visual cortices. In the mild case, phosphorylation was reduced in the right parieto-temporal area. In another dynamic FDG-PET study of seven patients with mild AD and six normal age-matched controls, Mosconi et al. (2007b) showed significant reductions in *K*_1_ in the hippocampus in AD compared to controls, although relative *CMR*_glu_ was better able to identify AD from controls, likely due to the additional contribution of reduced glucose phosphorylation.

A small number of tracer studies have been performed in rodents, which all support reduced transport of glucose in AD. Do et al. (2014) perfused brains of 3xTgAD and wild-type mice with [3H]-D-glucose (0.3 mCi/ml) immediately prior to decapitation and measurement of tissue radioactivity using a scintillation counter (Do et al., 2014). No difference in [3H]-D-glucose uptake was found in 3xTgAD mice compared to wild-types aged 6 or 8 months, but a significant decrease was found in 3xTgAD mice at 18 months. The reduced uptake at 18 month was associated with reduced expression of GLUT1 at the same timepoint. Ding et al. (2013) used micro-FDG-PET to study glucose uptake in 3xTgAD mice and wild-types at various ages. FDG-PET signals were measured at 40 min post injection of FDG, and are likely to reflect both transport and utilization. Reductions in the FDG-PET signal were found in both AD and wild-type animals with age, but while no formal comparison was made, FDG-PET signals did not appear to differ across genotype. Last, Merlini et al. (2011) performed microdialysis in arcAβ following intravenous injection of glucose and found reduced uptake compared to wild-types, despite increases in IgG extravasation in AD mice.

### Effects of GLUT Alterations on Brain Physiology

The effects of GLUT disruptions on the brain have been studied in rodent models. Abdul Muneer et al. (2011) induced GLUT1 disruption in a mouse model through the administration of methamphetamine. This led to a reduction in BBB tight junction proteins, indicating that GLUTs may play a role in regulating BBB integrity. Winkler et al. (2015) later demonstrated that GLUT1 deficient mice overexpressing APP showed changes characteristic of AD. These included reduced brain capillary levels of low-density lipoprotein receptor-related protein 1 (LRP1), a transporter at the BBB which clears Aβ from the brain (Zlokovic, 2008, 2011), diminished cerebral blood flow, early BBB breakdown, accelerated Aβ deposition in the hippocampus and cortex, neuronal dysfunction and cognitive impairment. They also observed that vascular changes preceded neuronal dysfunction in these mice. Decreased levels of GLUT1 and GLUT3 were found in a rat model of sporadic AD, achieved through the intracerebroventricular injection of streptozotocin, alongside impaired insulin signaling and abnormalities in phosphorylation and microtubule binding activity of tau (Deng et al., 2009).

Effects of severe glucose transporter depletion on early brain development can be observed in human GLUT1 deficiency syndrome, a rare genetic disorder characterized by impaired glucose metabolism due to a deficiency in GLUT1. Clinical features include intellectual disability, movement disorders and epileptic seizures refractory to treatment. Late-onset GLUT1 deficiency syndrome affects children at an older age, with evidence showing mild to moderate intellectual disability (Leen et al., 2010). A later study by the same group followed up patients with GLUT1 deficiency syndrome between 18 and 41 years old. Their results showed that while the prominent feature during childhood is epilepsy, this diminishes later in life and new movement disorders become apparent during adolescence. Cognitive function, however, did not appear to worsen with age (Leen et al., 2014). There is no evidence of GLUT1 deficiency syndrome manifesting in late adulthood.

### Links Between Insulin Resistance and GLUT Changes

Deficits in transport and metabolism in AD may result from impaired insulin signaling, particularly due to alterations in the function of insulin-sensitive transporters. Mullins et al. (2017) investigated the relationship between the pathological hallmarks of AD and genes related to insulin resistance by converting histological and gene expression data into 3D spatial maps. Their findings showed that GLUT1 was positively correlated, whereas GLUT4 was negatively correlated, with insulin signaling proteins (including IRS-1).

In a mouse model of AD, Chua et al. (2012) found that insulin signaling molecules were increased, alongside increases in GLUT3 and GLUT4 expression and decreases in brain insulin and glucose content, and that these changes occurred earlier than pathological accumulation of Aβ. In the STZ rat model of AD, increases in GLUT2 were accompanied by decreases in insulin receptors (Knezovic et al., 2017). In the same model, Deng et al. (2009) found decreases in insulin signaling alongside decreases in GLUT1 and GLUT3 (Deng et al., 2009).

Insulin resistance has been targeted as a means to restore glucose transport by number of groups. In a small randomized control trial, Gejl et al. (2017) examined the effects of GLP-1 analog treatment in patients with a clinical diagnosis of AD. In their study, 18 participants received liraglutide and 20 received placebo in a 26-week period. Their results showed that treatment with liraglutide significantly raised the average blood-brain glucose transport capacity estimate (*p* < 0.0001) from 0.72 to 1.1 (μmol/g/min) compared to placebo, which positiviely correlated with measures of cognition. Salkovic-Petrisic et al. (2014) performed long-term dosing of STZ rats with galactose as an alternative energy source to glucose and found upregulation of GLUT3 transporters back to levels observed in healthy rats and improved cognitive deficit.

## Discussion

This systematic review examines the evidence for alterations in glucose transport in AD and the effect of these alterations on the brain. The majority of studies investigating GLUT1 and GLUT3 suggest that both transporters are reduced in the hippocampus and cortex of AD brains. Longitudinal rodent studies did not find changes at early timepoints, but consistently observed reductions in transporter expression after Aβ pathology had developed, indicating that Aβ itself may be responsible.

There were fewer studies investigating other GLUTs including GLUT2, GLUT4, GLUT12, and GLUT14. A rise in GLUT2 in AD brain tissue was observed by Liu et al. (2009) and Knezovic et al. (2017). In the study by Liu et al. (2009), GLUT2 changes were exacerbated in patients who also had T2DM. It is possible that GLUT2 increases to serve as a compensatory mechanism of GLUT1 and GLUT3 loss. The role of GLUT2, therefore, requires further investigation. No changes in GLUT4, an insulin sensitive transporter, were found in aged NSE/hPS2m (Lee et al., 2013) or 3xTg mice (Griffith et al., 2019), whereas Chua et al. (2012) found increases in AβPPsw/PS1ΔE9 mice. Pujol-Gimenez et al. (2014) showed GLUT12, also an insulin-sensitive transporter, was increased in AD brain tissue. A study by Purcell et al. (2011) showed that overexpression of GLUT12 in healthy mice improved whole-body insulin sensitivity, indicating GLUT12 may play a compensatory role when insulin resistance develops in AD. The findings by Wang et al. (2012) on GLUT14 demonstrate that SLC2A14 polymorphisms may increase susceptibility to late-onset AD. Further work is required to determine the role of these lesser-studied GLUTs in AD and gauge their therapeutic potential.

The cause of glucose transporter expression in AD remains unclear. One suggestion for the reduction of GLUT1 in the AD brain is an abnormality in the translation process of the transporter. Brains of AD patients have been found to express low levels of transcription factor hypoxia-inducible factor 1α, a protein complex which regulates GLUT1 and GLUT3 expression (Liu et al., 2008; Cohen et al., 2014). However, Mooradian et al. (1997) did not observe any changes in GLUT1 mRNA concentrations, which suggests a post-translational abnormality. Another study proposed that the activation of calpain I, a calcium sensitive protease, is a potential cause of GLUT3 under-expression (Jin et al., 2013) due to its damaging effect through proteolysis of GLUT3 promoters. Calpain I has also been associated with downregulation of protective mechanisms against tau phosphorylation (Gu et al., 2018).

AD pathologies such as Aβ or tau may directly reduce GLUT expression. The low capillary density found within Aβ plaques in the study by Kawai et al. (1990) may suggest that Aβ plaques lead to the degeneration of capillaries, which may in turn affect GLUT expression. Findings by An et al. (2018) suggest that a reduction of GLUT3 but not GLUT1 is more closely correlated with Aβ and NFT abnormalities. Studies using amyloidogenic mouse models support a link between spatial proximity of Aβ to glucose transporter alterations (Kouznetsova et al., 2006; Kuznetsova and Schliebs, 2013), and indicate that GLUT changes occur after development of Aβ pathology (Hooijmans et al., 2007; Kuznetsova and Schliebs, 2013; Do et al., 2014; Griffith et al., 2019). Injection of Aβ_1−42_ into the brain of healthy mice produced very similar effects on GLUT expression as observed in AD mice, indicating Aβ plays a direct role in modulating GLUT expression (Gil-Iturbe et al., 2020). Mark et al. (1997) examined the relationship between Aβ and glucose transport, by examining cultured rat hippocampal and cortical neurons. Their results demonstrate that Aβ deposition impairs glucose transport through a mechanism involving membrane lipid peroxidation. The authors suggest that this mechanism may further lead to neurodegeneration in AD. In a drosophila study, Niccoli et al. (2016) demonstrated a protective effect against Aβ toxicity by genetically upregulating glucose transporters. Similar results were demonstrated when using metformin, an anti-diabetic drug designed to stimulate glucose uptake into cells. Human studies are yet to confirm these findings.

Without measuring glucose uptake into brain tissue, it is difficult to know how changes in GLUTs affect glucose availability in parenchymal cells. Tracer studies that measure uptake of glucose and glucose-analogs into brain are therefore crucial for determining the impact of GLUT changes on glucose transport. Uptake of glucose can be measured using a range of methods from invasive perfusion-based approaches, to non-invasive imaging techniques such as FDG-PET. Generally, tracer-based studies performed to date show that transport of glucose into the brains of AD patients and rodent models of AD is reduced. Unfortunately, these studies cannot distinguish between glucose transport through the BBB or through cell membranes. The affinity of glucose for GLUT3 (*K*_t_ = 1.4 mM, measured using 2DG) is much higher than that of GLUT1 (*K*_t_ = 6.9 mM, also measured using 2DG; lower *K*_t_ values relate to higher affinity), and therefore glucose transport across the BBB is likely to be rate limiting under most conditions (Simpson et al., 2008). Furthermore, Lund-Andersen (1979) and Pardridge (1994) argue that because the total surface area and mass of GLUTs on parenchymal cells far exceeds that of endothelial cells, glucose uptake into the intracellular space is limited by GLUT1 at the BBB, not by astrocyte GLUT1 or neuronal GLUT3. Therefore, it is likely that reductions in uptake of glucose or glucose-analogs into brain tissue reflect mainly reductions to GLUT1 at the BBB.

GLUT1 and GLUT3 are considered insulin-independent glucose transporters, and therefore are assumed not to be affected by insulin or insulin-like growth factors. It is therefore expected that insulin-resistance does not alter uptake of glucose through these transporters. However, there is some evidence that GLUT1 and GLUT3 may be moderately affected by insulin. Hernandez-Garzón et al. (2016) observed that astrocytic insulin-like growth factor receptor modulates the expression of GLUT1, but the authors did not determine if this affected uptake of glucose. Muhič et al. (2015) did not observe any effects of insulin or insulin growth factor on glucose uptake into astrocytes, suggesting that GLUT1, and even the insulin-sensitive transporter GLUT4 present on astrocytes, are not particularly sensitive to insulin. The effects of insulin on endothelial GLUT1 has not been studied. Hypoglycemia is known to cause upregulation of BBB GLUT1 and neuronal GLUT3, an adaptive mechanism to ensure sufficient glucose is delivered to the brain (Kumagai et al., 1995; Simpson et al., 1999; Yun et al., 2018). It is possible these changes occur due to hyperinsulinemia—a study in man showed that insulin increases uptake of glucose across the BBB, however the dose of insulin required to detect an effect was non-physiological. It is likely the dominant driver of GLUT1 changes at the BBB is glucose itself; *in-vitro* studies show hyperglycemia increases GLUT1 and glucose uptake in the absence of insulin (Takakura et al., 1991; Sajja et al., 2014). Despite this, the studies of Deng et al. (2009), Chua et al. (2012), and Mullins et al. (2017) support an association between insulin resistance and altered expression of insulin-insensitive transporters GLUT1 and GLUT3.

It is possible the link between insulin resistance and alterations to insulin-insensitive GLUTs occurs via a joint relationship with AD pathologies Aβ and tau. Mullins et al. (2017) found areas exhibiting lower expression of glucose transporters and insulin signaling genes had higher levels of Aβ and tau pathology. Insulin is known to promote Aβ clearance (Watson et al., 2003) while insulin resistance promotes the formation of Aβ oligomers (Yamamoto et al., 2012). It is suggested that a positive feedback loop subsequently occurs as Aβ oligomers lead to increased phosphorylation of insulin signaling proteins (Yoon et al., 2012) which, in turn, leads to further insulin resistance. The results from the randomized controlled trial on the use of liraglutide in AD provides further insight into the possible link between insulin resistance, glucose transporters and AD (Gejl et al., 2016). Their results show an improvement in glucose transport capacity with liraglutide. It is not clear whether this improvement reflects an increase in glucose transporters or an increase in postprandial insulin levels, however, as liraglutide does not cross the BBB, it is possible that it causes a direct effect on the BBB itself.

## Clinical Significance and Future Perspective

Altered glucose metabolism occurs several years before evidence of cognitive impairment in AD (Chen and Zhong, 2013). Altered glucose transport has also been observed in mild cognitive impairment (MCI) (Mosconi et al., 2013), and deletion of glucose transporters has been shown to cause substantial neurodegenerative effects in animal models (Nicholson et al., 2010; Ding et al., 2013; Winkler et al., 2015). This suggests that drugs targeting the restoration of normal GLUT expression may be highly effective at reducing cognitive decline and transition from MCI to AD. Only a small proportion of drugs are able to cross the BBB, mainly due to the characteristic properties of desolvation, lipophilicity, molecular volume and dipole moment required for molecules to cross the BBB (Fong, 2015). Additionally, the BBB has tight control over what molecules leave the brain, making drug development in neurodegenerative disease more challenging (Pardridge, 2009). GLUT1 therefore becomes a highly attractive therapeutic target since it is present on the BBB itself.

GLUTs play a significant role in AD pathology with substantial evidence suggesting that GLUT1 and GLUT3 reductions occur following amyloid accumulation, but may precede the onset of clinical symptoms, while GLUT2 and GLUT12 appear to increase and may have a compensatory role. FDG-PET imaging could provide a means to detect reduced glucose transport in a clinical setting. Repurposing anti-diabetic drugs shows promising results in human studies of AD (Gejl et al., 2016, 2017). With evidence suggesting that metabolic changes can accurately predict subsequent cognitive decline (De Leon et al., 2001; Jagust et al., 2006; Mosconi et al., 2008), therapeutic strategies aiming to modify glucose transport could have a significant impact in clinical management of AD.

## Data Availability Statement

The original contributions presented in the study are included in the article/supplementary material, further inquiries can be directed to the corresponding author/s.

## Author Contributions

NK, BD, HE, LP, and OS contributed to the conception and design of the study. NK wrote the first draft of the manuscript. All authors contributed to manuscript revision, read, and approved the submitted version.

## Conflict of Interest

The authors declare that the research was conducted in the absence of any commercial or financial relationships that could be construed as a potential conflict of interest.
